# Does Gluten-Free Diet Protect Children with Celiac Disease from Low Bone Density?

**Published:** 2014-07-04

**Authors:** Merve Usta, Nafiye Urganci

**Affiliations:** Department of Pediatric Gastroenterology, Sisli Hamidiye Etfal Education and Research Hospital, Istanbul, Turkey

**Keywords:** Celiac, Gluten Free Diet, Low Bone Density, Bone Mineral Density

## Abstract

***Objective:*** We aimed to assess the effect and duration of gluten-free diet on bone health in children with celiac disease in our study.

***Methods:*** Sixty three patients with celiac disease (CD) formed the study group. They were divided into two subgroups according to their dietary compliance. Bone mineral density (BMD) values of the patients at two and five years of gluten-free diet (GFD) were determined.

***Findings*** ***:*** The relationship between BMD and compliance to GFD was found to be statistically significant (*P*<0.01). BMD z-scores were increased (0.12±0.15 and 0.10±0.14 units respectively) (*P*<0.01). The patients in group 1 and 2 had mean -1.18±0.83 and -2.06±0.73 z-scores in the first DXA. In the second DXA, these values were -1.10±0.73 and-1.94±0.93 respectively.

***Conclusion:*** Dietary compliance is important for bone health, and the time needed to normalize the BMD is not known. Patients with positive anti-endomysium antibody (EMA), poor dietary history and history of bone pain should be evaluated with DXA during follow-up.

## Introduction

Celiac disease (CD), a common cause of malabsorption in childhood, is an abnormal immuno-mediated response to the ingestion of gluten and other peptides from different cereals (wheat, barley, and rye) in genetically susceptible subjects. Life-long withdrawal of gluten from diet is the treatment of CD^[^^1^^]^. Defective absorption of calcium and vitamin D secondary to small intestinal mucosal damage, secondary lactose malabsorption, increased endogenous calcium use, fecal loss, and impaired vitamin D absorption and pro-inflammatory cytokines may predispose derangements of bone and mineral metabolism in patients with CD. Skeletal diseases such as rickets, osteomalacia or osteoporosis may be observed in patients with CD^[^^2^^,^^3^^]^. Compliance with a gluten-free diet (GFD) reverses the histological changes in the intestine and also the biochemical evidence of calcium malabsorption^[^^4^^]^.

 During the recent years, many studies were conducted relating to calcium and vitamin D levels and bone health in children with celiac disease. Dual-energy x-ray absorptiometry (DXA) is a method which is simple, low-cost and frequently used for assessment of bone health in children^[^^5^^]^. In this context, the studies using DXA showed that bone mineral density (BMD) was low in untreated celiac patients, and this was resolved with gluten-free diet^[^^2^^,^^6^^,^^7^^]^. However, some subjects still could not be clarified; e.g. how many years of gluten-free diet will be needed to improve BMD? The age at diagnosis may be important; because older children may show slower improvement^[^^8^^]^. GFD improves bone mineral density but does not normalize it in all patients^[^^3^^]^. But children on GFD are not strictly at increased risk for low bone mineral density (BMD)^[^^9^^]^.

 In the present study, we aimed to assess the effect and duration of gluten-free diet on bone health in children with celiac disease.

## Subjects and Methods

One hundred twenty eight children (53 boys, 75 girls, mean age 13.2±5.09 years), diagnosed with CD based on the revised criteria of the European Society for Pediatric Gastroenterology, Hepatology, and Nutrition^[^^10^^]^ were followed-up at Sisli Hamidiye Etfal Education and Research hospital department of pediatric gastroenterology outpatient clinic between 1999 and 2012. These children were monitored through periodic visits (every 3-6 months) for assessment of symptoms, growth, physical examination and compliance with GFD.

 The patients who had been on GFD for at least two years, had no evidence of height and weight less than 3rd or more than 97th percentile, and had no evidence of precocious or delayed puberty (For girls normal puberty was defined as breast development beginning between 8 and 13 years of age. For boys, the criteria were testes size of at least 4 ml between 9 and 14 years of age) were retrospectively included in the study.

 The patients who had other disease(s) known to affect BMD (chronic inflammatory diseases, endocrine disturbances, history of childhood cancer, or prior transplantation, chronic liver or kidney disease), received calcium, hormone or dietary supplements except for iron, and had been on GFD for less than 2 years were excluded from the study.

**Fig. 1:**
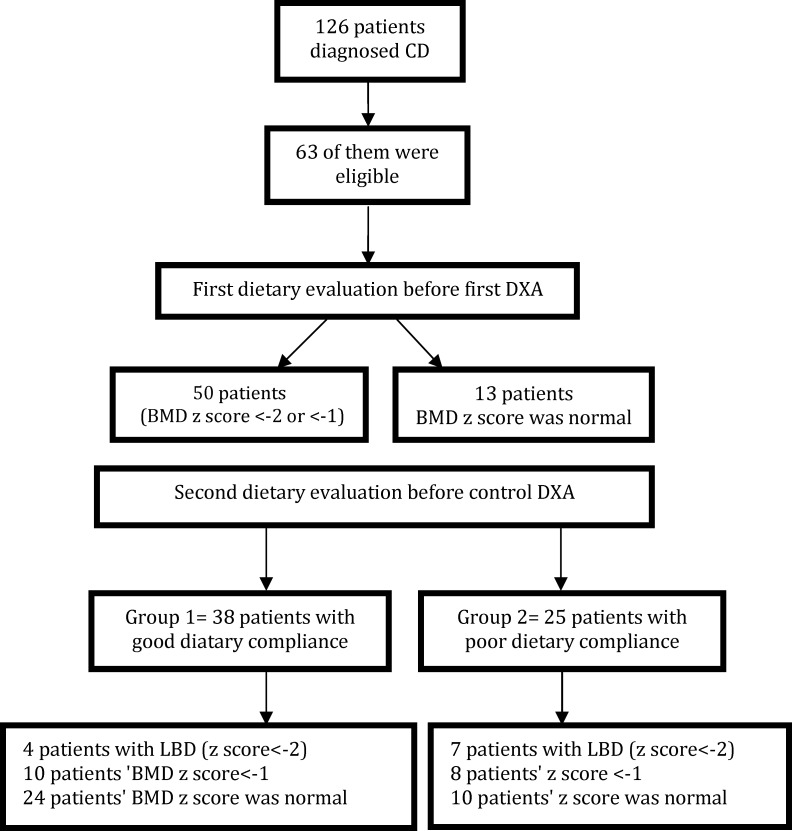
Flowchart of the patients in the study

 Sixty three patients were found eligible. The flowchart of the patients is shown in Fig. 1. Demographic data (sex, date of birth) and disease characteristics (age at diagnosis, disease duration, GFD duration) were recorded for every subject. Subjects were screened through their medical records, and eligibility was confirmed by physical examination. Consent was obtained from subject’s parent or guardian. The study group included 27 boys and 36 girls (mean age 14.66±5.05 years). All patients in our study had been on GFD for 2 years when the first DXA was performed.

 Dietary compliance to GFD was assessed twice. First assessment was performed using DXA before the first BMD measurement. The second dietary assessment was performed before the second DXA. The study group was divided into two subgroups according to the dietary compliance during the last 6 months. Group 1 (38 children) included the patients who were strictly on GFD. The patients with poor compliance to GFD were included in group 2 (25 children). Compliance with the diet was ascertained by anti-endomysium antibodies and three-day dietary history before visit to outpatient clinic, and by clinical condition during follow-up period. The patients who had positive anti-endomysium antibodies and were non-compliant with the diet as found out from their dietary history during the outpatient clinic visit were included in group 2. The second DXA was performed after 5 years of GFD (3 years after the first DXA).

***Bone mineral measurements:*** BMD values at lumbar 2-4 vertebrae and femoral neck were measured by dual-energy x-ray absorptiometry (DXA, Lunar, DPX 5784 software system, version 4.6). BMD was expressed in grams per square centimeter (gr/cm²), and z-score was used in this study because it eliminates age and sex by considering each patient’s BMD with respect to the age- and sex-matched normal population. Low bone mineral content or bone mineral density is defined as a BMC or areal BMD z-score that is less than or equal to -2.0, adjusted for age, gender and body size, as appropriate according to the 2007 guidelines of the International Society for Clinical Densitometry (ISCD)^[^^11^^]^.

***Biochemical measurements:*** Serum calcium (normal range 8.8–10.8 mg/dL), phosphate (normal range 2.7–4.5 mg/dL), alkaline phosphatase (reference value 250–1000 IU/L) and magnesium (normal range 1.7-2.3 mg/dL) were determined in two celiac groups using standard methods (radioimmunoassay (RIA, Diasorin Inc., Stillwater, MN).

Intact parathormone levels (normal range: 12–72 pg/mL) were determined by chemiluminescence method (Immulite, Bio-DPC, Los Angeles, CA) in both groups.

***Statistics:*** NCSS (Number Cruncher Statistical System) 2007 and PASS (Power Analysis and Sample Size) 2008 Statistical Software (Utah, USA) software were used for statistical analysis. Data were evaluated with descriptive statistical methods (mean, standard deviation, frequency, ratio) and paired samples test was used for the evaluation of control measurements compared with the first measurements. Mann-Whitney U test was used to evaluate BMD z-scores according to dietary compliance. chi-square test was used to compare the qualitative data. The results were considered to be significant at a level of *P*<0.05 (95% confidence interval).

## Findings

First evaluation with DXA was performed at two years of GFD. Low bone density (BMD z-score less than or equal to -2) or z-score <-1 was found in 50 patients (79.4 %). DXA results were normal in 13 patients (20.6%) in the first DXA. Compliance to GFD was evaluated using EMA, and dietary history during the last three days was noted on medical records of the patients during the first DXA. Compliance to gluten-free diet was good in twelve patients and poor in one patient among the children with normal DXA (n=13). Low bone density (LBD) was found in 21 (42%) patients whose dietary compliance was good and in despite the 29 (38%) patients whose dietary compliance was poor. The relationship between low bone density and poor dietary compliance was found to be statistically significant (*P*<0.01). 

**Table 1:** BMD of the patients in the first and the second DXA in the study group

**Variable**	**First DXA**	**Second DXA**	***P. *** **value** *
	**Mean (SD) **	**Mean (SD) **	
**BMD L2-L4 (gr/cm** ^2^ **)**	0.74 (0.15)	0.87 (0.15)	0.001
**BMD Femur neck (gr/cm** ^2^ **)**	0.74 (0.12)	0.84 (0.15)	0.001
**Z-Score (L2-L4) **	1.68 (1.14)	1.40 (0.84)	0.2

SD: Standard deviation

* Paired Samples Test; BMD: bone mineral density; DXA: Dual-energy x-ray absorptiometry;

 The mean concentrations of calcium, phosphate, alkaline phosphatase, magnesium and iPTH were within the normal limits during the first DXA.

 The second DXA was performed 3 years after the first DXA in the study group. In Table 1, BMD values of the patients after two years of GFD (first DXA) and after five years of GFD (second DXA) are compared. The first BMD of lumbar vertebrae and femoral neck was increased (0.12±0.15 and 0.10±0.14 units respectively) compared to the control BMD, and these increases were found to be statistically significant (*P*<0.01).

 Two subgroups were formed according to the dietary compliance before the second evaluation of BMD with DXA. Patients in group 1 and 2 were compared according to z-score at lumbar vertebrae (Table 2). The patients in group 1 had a mean z-score of -1.18±0.83 at lumbar vertebrae in the first DXA. Patients in group 2 (poor GFD compliance) had a mean z-score of -2.06±0.73 at lumbar vertebrae in the first DXA. In the second DXA, the mean scores were -2.50±073 and -1.94±0.93 respectively. The differences were significant (*P*=0.03 and *P*=0.047). Table 3 and 4 show the celiac patients according to z-scores at L2-4 in group 1 and 2. In the first DXA, the difference was significant. In the control group, there still were seven patients whose BMD values were <-2 in group 2, and 14 patients in group 1 (BMD <-2 or -1) had abnormal DXA results at the second DXA but the difference was not statistically significant.

**Table 2:** z score (L2-L4) of the patients in group 1 and group 2 at the first and second DXA

	**Group 1**			**Group 2**			***P. *** **value** *
	**Mean**	**SD**	**Median**	**Mean **	**SD **	**Median**	
**First DXA**	1.18	0.83	1.12	2.06	1.16	2.10	0.003
**Second DXA**	2.50	0.73	1.05	1.94	0.93	1.85	0.047

* Mann Whitney U test; DXA: Dual-energy x-ray absorptiometry; SD: Standard deviation

## Discussion

Low bone mineral density is common in children with celiac disease. Several studies documented bone mineral alterations at the time of diagnosis in children with CD^[^^2^^,^^6^^,^^8^^]^. Some longitudinal and cross-sectional studies reported variable but remarkable results for improvement of bone mass during GFD consumption^[^^2^^,^^6^^,^^8^^,^^9^^,^^12^^,^^13^^]^. In adult celiac patients, GFD was found to improve, but not normalize bone mass ^[^^13^^]^. Some studies on children report that bone mineral density was normalized 1 year after starting GFD^[^^6^^,^^14^^]^. On the contrary, in other studies, normal standards could not be reached although significant improvements were seen with GFD^[^^2^^,^^8^^]^. 

**Table 3:** The value of z score at L2-L4 in both groups at the first DXA.

**Group**	**BMD z score< -2**	**BMD z score< -1**	**Normal **	***P. *** **value**
**Group 1**	10 (26.3 %)	16 (42.1 %)	12 (31.5 %)	0.01
**Group 2**	14 (56 %)	10 (40 %)	1 (4 %)	

* Chi-square test; DXA: Dual-energy x-ray absorptiometry

**Table 4:** The value of z sore at L2-L4 in both groups at the control DXA

**Groups**	**BMD z score< - 2**	**BMD z score< -1**	**Normal **	***P. *** **value** *
**Group 1**	4 (10.5 %)	10 (26.3 %)	24 (63.1 %)	0.1
**Group 2**	7 (28 %)	8 (32 %)	10 (40 %)	

* Chi-square test; DXA: Dual-energy x-ray absorptiometry

In the study conducted by Kalayci et al^[^^2^^]^, the authors reported that although their patients’ BMD values were significantly increased after one-year treatment, 37.5% of the patients on GFD for a mean period of 40.4 months were osteopenic. Similar to the mentioned studies, the BMD z-scores were less than -2 or -1 in 79.4% of the patients by DXA at the second year of the diet. The values of 13 patients were normal. 

 In a study, the authors found spine and whole-body BMD of children with celiac disease to increase 0.06 g/cm^2^ and 0.05 g/cm^2^, respectively, after 1.4±0.4 years on GFD. According to these authors, BMD was normal at the end of the study^[^^15^^]^. Tau et al^[^^12^^]^ reported that some patients showed recovery of bone mass after only 3–7 months of being on GFD, whereas others only showed recovery after 1.5 years of therapy. In the present study, the second DXA was performed 3 years (mean period) after the first DXA, and improvement of the BMD z-scores, the values were less than -2 or -1 in 29 patients. Some authors recommend that BMD should be measured at the diagnosis and after a year of strict gluten-free diet in celiac with malabsorption symptoms^[^^3^^]^. But if ayear is sufficient to normalize bone mineralization or where this does not happen, when it will be necessary to start a supplementation is not clear. In the present study, we aimed to evaluate DXA not at diagnosis, but after 2 years and 5 years (mean) of GFD. In the first DXA, the results of 13 patients were normal. In the second DXA, the BMD z-scores of 29 patients were less than -2 or -1. Although we observed an improvement in the BMD values after 5 years of GFD, the results were not completely normalized in all of our patients.

 Patients with CD are at increased risk of fractures, particularly in the peripheral skeleton^[^^16^^]^. A recent meta-analysis demonstrated that the pooled odds ratios for all fractures in CD patients was 1.43 compared with control group^[^^17^^]^. In the present study, although no fractures were observed in any of the patients, low bone density is risky in this respect. It is still not clear when to request DXA in the follow-up of children with celiac disease. In our study, good compliance to gluten-free diet improved the bone density levels, but there still were patients whose values could not be normalized after 5 years.

 Blazina et al^[^^9^^]^ found that good compliance to gluten-free diet is mandatory to maintain optimal bone health. Children and adolescents on not strict GFD are at increased risk for low BMD. The authors also recommend that BMD should be evaluated in patients with positive EMA^[^^9^^]^. It is quite difficult to evaluate the compliance to GFD in children. They may unintentionally be exposed to gluten^[^^8^^,^^18^^]^. In the present study, dietary compliance was evaluated by EMA-positivity during follow-up and by the three-day diet list brought before admission to the outpatient clinic. Based on the results of both DXAs, we found a statistically significant relationship between compliance to GFD and improvement in BMD. The limitation of our study was that we made the evaluation retrospectively using the three-day dietary history on the file records. Another limitation was that we did not investigate the bone resorption markers. These markers are not routinely used since they are not tested in practice everywhere although they may be more useful in evaluation of bone health^[^^8^^]^.

 In another study, the authors concluded that celiac children not following GFD show delayed bone maturation and mineralisation^[^^7^^]^. Poor dietary compliance affected bone health negatively in our study. We also observed that BMD z-score was not normal in fourteen patients in group 1 at the fifth year of GFD. We think that strict adherence to gluten-free diet affects BMD positively but duration of GFD needed to normalize BMD remains unclear.

## Conclusion

Dietary compliance is an important factor for bone health in children with celiac disease. Children diagnosed with celiac disease based on positive EMA, poor dietary history as well as signs of bone pain should be re-assessed with DXA, and information and consideration should be given about mineral-active drugs during the follow-up period.
